# Beta-Blockers for Primary Prevention of Anthracycline-Induced Cardiac Toxicity: An Updated Meta-Analysis of Randomized Clinical Trials

**DOI:** 10.1155/2022/8367444

**Published:** 2022-12-29

**Authors:** Armin Attar, Arman Karimi Behnagh, Mehrasa Hosseini, Foad Amanollahi, Paria Shafiekhani, Ali Kabir

**Affiliations:** ^1^Department of Cardiovascular Medicine, TAHA Clinical Trial Group, Shiraz University of Medical Sciences, Shiraz, Iran; ^2^Minimally Invasive Surgery Research Center, Iran University of Medical Sciences, Tehran, Iran; ^3^Student Research Committee, Shiraz University of Medical Sciences, Shiraz, Iran; ^4^Functional Neurosurgery Research Center, Shohada Tajrish Comprehensive Neurosurgical Center of Excellence, Shahid Beheshti University of Medical Sciences, Tehran, Iran

## Abstract

**Aim:**

Cardiotoxicity is a well-recognized complication of chemotherapy with Anthracyclines. However, results from trials evaluating beta-blockers for prevention are controversial. Therefore, we performed a meta-analysis to find whether prophylactic administration of beta-blockers can help prevent Anthracyclines-induced cardiotoxicity.

**Methods:**

We assessed randomized trials and observational studies where a prophylactic intervention was compared with a control arm in patients with a normal left ventricular ejection fraction (LVEF) receiving Anthracyclines. The primary outcome was EF reduction. The secondary outcome was the development of Cancer Therapeutics–Related Cardiac Dysfunction (CTRCD), defined as a decrease in the LVEF of >10% to a value of <53%.

**Results:**

We included 17 trials comprising 1291 patients (671 patients in the intervention arm and 620 in the control arm). Carvedilol was administered in eight studies, and others used bisoprolol, metoprolol, or nebivolol. Compared with baseline, LVEF reduced in both intervention and control groups after chemotherapy (MD = −1.93%, 95% CI: -2.94, -0.92, *p* = 0.001, I^2^ = 72.1% vs. MD = −4.78%, 95% CI: -6.51, -3.04, *p* = 0.001, *I*^2^ = 91.6%, respectively). LVEF was less reduced among the beta-blocker receivers (MD = 3.44%, 95% CI: 1.41–5.46, *p* = 0.001, I^2^ = 94.0%). Among the eight studies reporting the incidence of CTRCD, 45 out of 370 participants in the intervention arm and 54 out of 341 in the control arm were reported to experience this complication (RR = 0.76; 95% CI: 0.53,1.09; *I*^2^ = 24.4%; *p* = 0.235).

**Conclusion:**

Treatment with beta-blockers prevents dilatation of the left ventricle, development of diastolic dysfunction, and reduction of LVEF. However, these hemodynamic effects do not translate into a significant reduction in CTRCD incidence and prevention of hospitalization for heart failure or cardiac death.

## 1. Introduction

Treatment with anthracyclines, a potent family of antineoplastic agents, has increased the survival rate of many cancerous patients, especially those with breast cancer and hematologic malignancies. However, this effect is at the expense of dose-related cardiotoxicity [1]. The incidence of heart failure (CHF) increases with the cumulative doxorubicin doses, and a dosage of 400, 500, and 550 mg/m^2^ results in 5%, 16%, and 26% CHF incidence rates, respectively [2]. Several strategies for the primary prevention of anthracycline-induced cardiotoxicity have been implemented. These have mainly focused on either the reduction of cardiotoxicity potency (using a less cardiotoxic derivative, continuous infusion, or liposomal encapsulation) or the administration of cardioprotective agents (dexrazoxane, angiotensin-converting enzyme inhibitors, angiotensin receptor blockers, and/or beta-blockers) [3, 4]. Beta-blockers are the most widely studied medications for the primary prevention of Cancer Therapeutics–Related Cardiac Dysfunction (CTRCD). While the first randomized clinical trial (RCT) on beta-blockers showed promising effects [5], the largest published data showed the contrary [6]. Therefore, performing a meta-analysis to find an answer to the observed controversies seems necessary.

The previous meta-analyses in the field were most outdated and as several new RCTs have been conducted since then, performing an updated meta-analysis seemed essential. Furthermore, most previous meta-analyses evaluated the hemodynamic effect of these medications on the heart, but the clinical translation of those findings needed to be clarified. Here, we have conducted an updated meta-analysis of RCTs focusing on the efficacy of beta-blockers for the primary prevention of CTRCD as an important clinical endpoint.

## 2. Materials and Method

### 2.1. Data Sources

**Figure 1:**
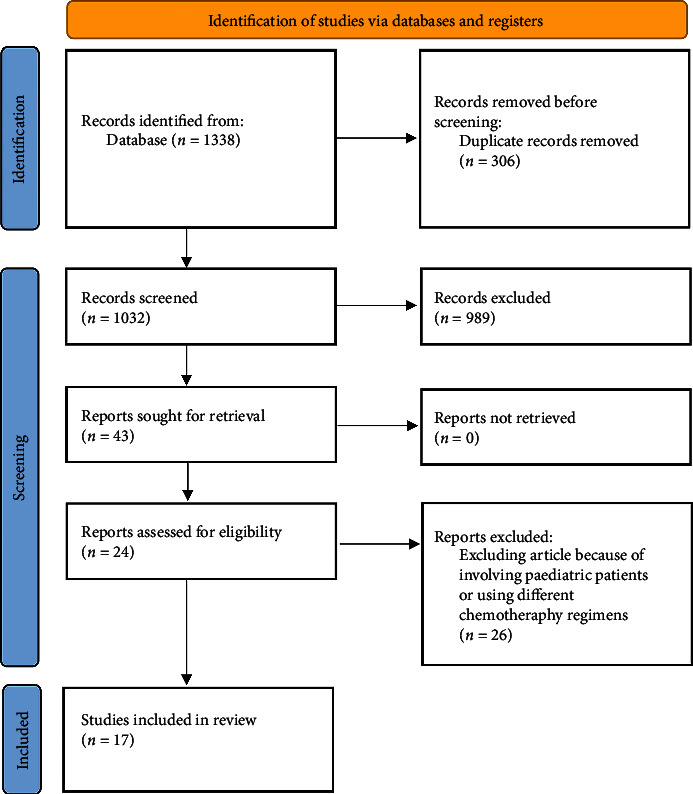
PRISMA flow diagram of study.

This meta-analysis was conducted using the PRISMA guideline [7]. The PRISMA Flow diagram of the study is shown in Figure 1. In addition, a systematic search of Scopus, PubMed, Web of Science, and Google Scholars was performed to identify the relevant studies. The timing of publication was disregarded as a factor in the mining process. The search was conducted using any combination of the following keywords: anthracyclines, cardiomyopathy, heart failure, carvedilol, and beta-blockers. Also, references mentioned in each article were explored to retrieve further possible candidates.

### 2.2. Study Selection

The inclusion criteria of the studies were as follows: (1) randomization in clinical trials, (2) study sample population older than 18 years of age with a diagnosis of malignancy at the time of enrolment, (3) beta-blocker administration earlier, simultaneous or after anthracycline therapy, although in a prophylactic setting, (4) a detailed explanation of the echocardiographic findings, with follow up periods lasting from 6 to 12 months.

### 2.3. Data Extraction and Bias Risk Assessment

The data extraction process was performed by independent reviewers and entered into an Excel sheet. Assessing the potential source of bias was performed using the Cochrane Collaboration tool [8]. Disagreements were discussed with a third-party reviewer, and the final results were obtained via consensus following the revision of the full-text articles. The information extracted from the selected articles were as follows: first author, publication forms (full text, abstracts, and letter), publication year, sample size, mean age, gender, type of malignancy, type of anthracycline used, accumulative anthracyclines dose, carvedilol dose, history of radiotherapy, and median follow-up duration. Extracted statistical data, subject to availability, included descriptive statistics for left ventricular ejection fraction (LVEF) pre/postanthracycline treatment and posttreatment cardiac events, other adverse events, comorbidities, OR, relative risk, 95% CI, and *p* values. The entirety of the data regarding outcomes other than the main outcome, such as echocardiographic findings (other than LVEF) and biomarkers related to heart disease, was also extracted. Clarification and further information on specific studies were obtained by directly contacting the corresponding authors of each study.

### 2.4. Outcomes

LVEF at varying endpoints, more commonly 6 months after chemotherapy, was this study's primary outcome of interest. Additional outcomes of interest included as follows: cardiac events such as LV dysfunction, cardiotoxicity, sudden cardiac death, and heart failure during the study and follow-up after anthracycline treatment. Secondary outcomes of interest about additional echocardiographic parameters and biomarkers reported heterogeneously across studies, at early and late follow-up periods were also included. Echocardiographic parameters consisted of global longitudinal strain (GLS), left ventricular end-diastolic diameter (LVEDD), left ventricular end-systolic diameter (LVESD), e's, E/e', isovolumic relaxation time (IVRT), and isovolumic relaxation time (IVCT). Moreover, an evaluation of cardiac biomarkers such as plasma brain natriuretic peptide (BNP), plasma myocardial enzymes, and troponin I level was performed in this study.

### 2.5. Statistical Analysis

Data pooling (or meta-analysis) was performed for each endpoint presented in two or more eligible studies. Sensitivity analysis was performed in the meta-analysis of LVEF to confirm the stability of the results, carried out by using STATA metaninf command. Subgroup analysis was performed for the study arms. Also, we represent the difference between the two arms of studies at the end of the primary end-point. Risk ratio (RR) was determined for the risk of adverse events, while the mean difference (MD) was used for the comparison of continuous indices. Results are presented with the corresponding 95% confidence interval (CI). Heterogeneity across each study was evaluated by Cochrane's Q statistic, and the *I*^2^ statistic was applied to quantify heterogeneity (*I*^2^ above 50% representing substantial heterogeneity). When the estimation of treatment effects was *I*^2^ < 50%, we used a fixed effect model; otherwise, the random-effect model was used. *p* values <0.05 were considered statistically significant. Harbord's and Egger's tests were conducted to assess the publication bias. Statistical analysis was performed using Stata software v. 14 (Stata CorpLP, College Station, TX, USA).

### 2.6. Patient and Public Involvement

There has been no patient and public involvement in this study.

## 3. Results

### 3.1. Search Results

In this investigation, we included 17 RCTs. A total study population of 1291 patients consisted of 671 patients in the intervention arm and 620 in the control arm. Among different types of beta-blockers, carvedilol was administered in eight studies [5, 6, 9–14], a combination of carvedilol with ACEI/ARB was used in 2 studies [15, 16], three studies considered metoprolol as preventive therapy [17, 18], nebivolol was used in two studies [19, 20], one study evaluated bisoprolol [21], and one study used the combination of lisinopril and bisoprolol [22]. The mean age of the participants was 48.5 ± 4.03 years (with a range of 38.4 to 54.4). The characteristics of the included studies are summarized in Table 1. It should be noted that both Heck et al. and Gulati et al. [23], studies were performed intervention assessments in the same population (PRADA trial at different time points. The data from the updated report by Heck et al. [18] was implemented for the majority of the outcome.

### 3.2. Primary Endpoint

**Figure 2:**
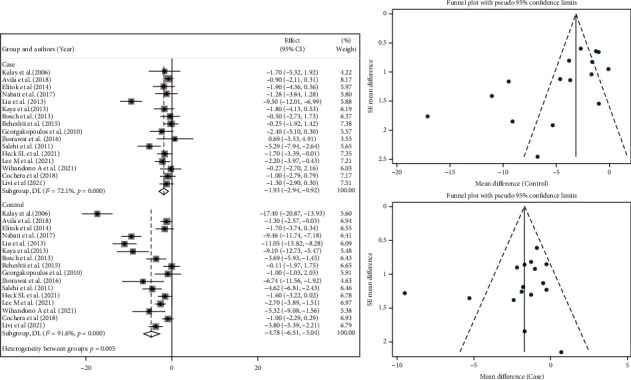
Forest and funnel plot of two group's LVEF changes at the end of studies.

Change in LVEF value, as the primary endpoint, was reported in all included studies. It was also reported after different durations of follow-up; however, it was measured in 7 studies after 6 months [5, 6, 9, 11, 13, 15, 19], in 3 studies after 4 months [12, 16, 22], and in four study after 12 months or more follow-up [14, 17, 18, 21]. The baseline and final mean value of LVEF of each included study are represented in Table 1. Pooling the data of all 16 studies represents a reduction in LVEF in both the intervention group and the control group after chemotherapy (MD = −1.93%, 95% CI: -2.94, -0.92, p = 0.001, *I*^2^ = 72.1% vs. MD = −4.78%, 95% CI: -6.51, -3.04, *p* = 0.001, *I*^2^ = 91.6%, respectively). Furthermore, comparing the LVEF change between both groups at the end of the studies showed that the LVEF was significantly less reduced among the beta-blocker receivers (MD = 3.44%, 95% CI: 1.41, 5.46; *p* = 0.001, *I*^2^ = 94.0%; Supplementary Figure 1). Besides, visual inspection of the funnel plot and statistical tests did not suggest any indication of publication bias for the included measuring LVEF (Figure 2; Egger's test *p* = 0.292).

**Figure 3:**
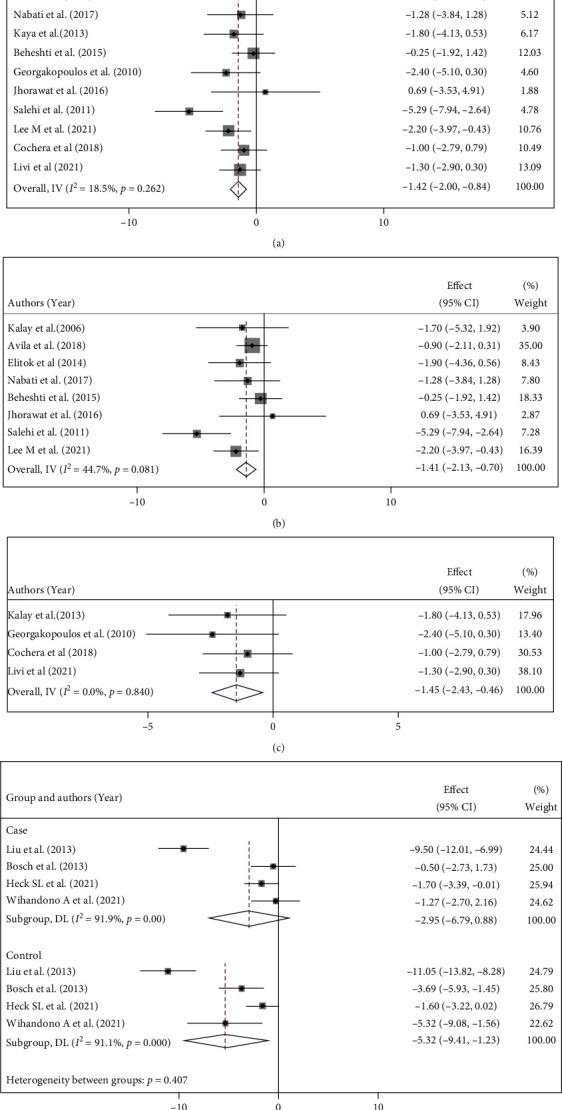
Forestplot of LVEF changes from the baseline of (a) Beta-blocker monotherapy and (b) Carvedilol monotherapy. (c) Other beta-blockers monotherapy. (d) Combination therapy.

Since different protective regimens were used, we performed a subgroup analysis regarding treatments. In this respect, Figure 3(a) represents data for studies that only used beta-blockers. Among them eight studies [5, 6, 9–14] used Carvedilol as the main therapy which resulted in MD = −1.41 (95% CI: -2.13, -0.70, *p* = 0.006, *I*^2^ = 44.7%; Figure 3(b)). Four studies [17, 19–21] used other beta-blocker monotherapies. The combination of these four studies revealed the same effects as we observed for the carvedilol monotherapy (MD = −1.45% (95% CI: -2.43, -0.46, *p* = 0.84, *I*^2^ = 0.0%; Figure 3(c)). Furthermore, four studies [15, 16, 18, 22] assessed the effect of the combination of beta-blockers with ACE or ARB. Pooling the findings of these four trials showed that the combination therapies had a lower reduction in LVEF compared to the corresponding control group in these studies (MD = −2.95%, 95% CI: -6.79, 0.88, *p* = 0.001, *I*^2^ = 91.9% vs. MD = −5.32, 95% CI:-9.41,-1.23, *p* = 0.001, *I*^2^ = 91.1%, Figure 3(d)).

### 3.3. Sensitivity Analysis

Among the included studies, some heterogeneities might affect the overall result. For instance, in the trials of Heck et al. and Livi et al., all the LVEF measurements were procured by CMRI and 3-dimensional echocardiography, respectively [18, 21]. Also, various protective regimens were implemented in different studies, from different types of beta-blockers to different combination therapies. The effect of monotherapy and combination therapy is shown in the previous section (Figure 3). Furthermore, other than factors associated with interventions, some parameters may bring diversity to the outcomes of each study. These factors were the heterogeneity in study populations. For example, in six studies, the participants had undergone radiotherapy before the study initiation [14, 15, 17–19, 21], and in Heck et al.'s study, in addition to radiotherapy, plenty of participants received trastuzumab [18]. By omitting the effect of these harmful features from the study population and the patients who received combination therapies, the LVEF in the intervention group changed to -1.22 (95% CI:-1.93,-0.50). In particular, to address each study's effect and the associated diversity on the meta-analysis, we used the metaninf command and the obtained results demonstrated in supplementary figure 2.

### 3.4. Secondary Endpoints

#### 3.4.1. Cardiomyopathy

For measuring the risk of CTRCD, we needed to use a decrease in the LVEF of >10 percentage points to a value of <53%. In this regard, eight studies reported the number of cases that showed a dramatic (more than 10%) reduction in LVEF [5, 6, 10–12, 15, 17, 21]. In these eight studies, 45 out of 370 participants in the intervention arm and 54 out of 341 participants in the control arm have been reported to experience this complication. There was an insignificant difference between the risk of 10% LVEF reduction between the beta-blocker receivers and placebo groups (RR = 0.76; 95% CI: 0.53,1.09; *I*^2^ = 24.4%; *p* = 0.235; Supplementary Figure 3). Moreover, publication bias was not observed for this outcome (P − harbord = 0.485).

#### 3.4.2. Mortality

Among all the included studies, 11 studies provided data on the number of deaths among the participants [5, 6, 9–12, 15, 17–20]. Based on the report of all these studies, in participants who had consumed beta-blockers (*N* = 475), only 16 participants died. The incidence of death among the controls (*N* = 445) was 26 cases. Also, of these studies, 4 reported that in their studies, none of the participants died during the study period. Moreover, Nabati et al. [11] reported a patient who died a few days after the initiation of the chemotherapy due to sepsis. Therefore, we did not include this case in our final analysis. Our fixed effect model showed that Beta-blocker consumption had been associated with lower mortality risk; however, this association was not statistically significant (RR = 0.58; 95% CI:0.34,1.02, p = 0.947, *I*^2^ = 0.0%, Supplementary Figure 3). Our analysis for addressing the possibility of publication bias revealed a lack of such bias for this estimate (P − harbord = 0.768).

#### 3.4.3. Hospitalization

Four studies reported the number of patients hospitalized during their study period [5, 6, 15, 19]. The result of our pooling demonstrated that the beta-blockers had no significant effect on reducing the risk of hospitalization among the patients (RR = 0.30 95% CI:0.50,1.98, *p* = 0.808, *I*^2^ = 0.0%, Supplementary Figure 3).

#### 3.4.4. Heart Failure

Among the included study, six studies provided details on the number of patients who developed heart failure during the studies [5, 6, 15, 17, 18, 21]. Our fixed effect model showed that despite the lower risk of heart failure development among the beta-blocker users, this association was not significant (RR = 0.33 95% CI:0.11,1.00, *p* = 0.998, *I*^2^ = 0.0%, Supplementary Figure 3).

#### 3.4.5. Diastolic Dysfunction Indices

**Figure 4:**
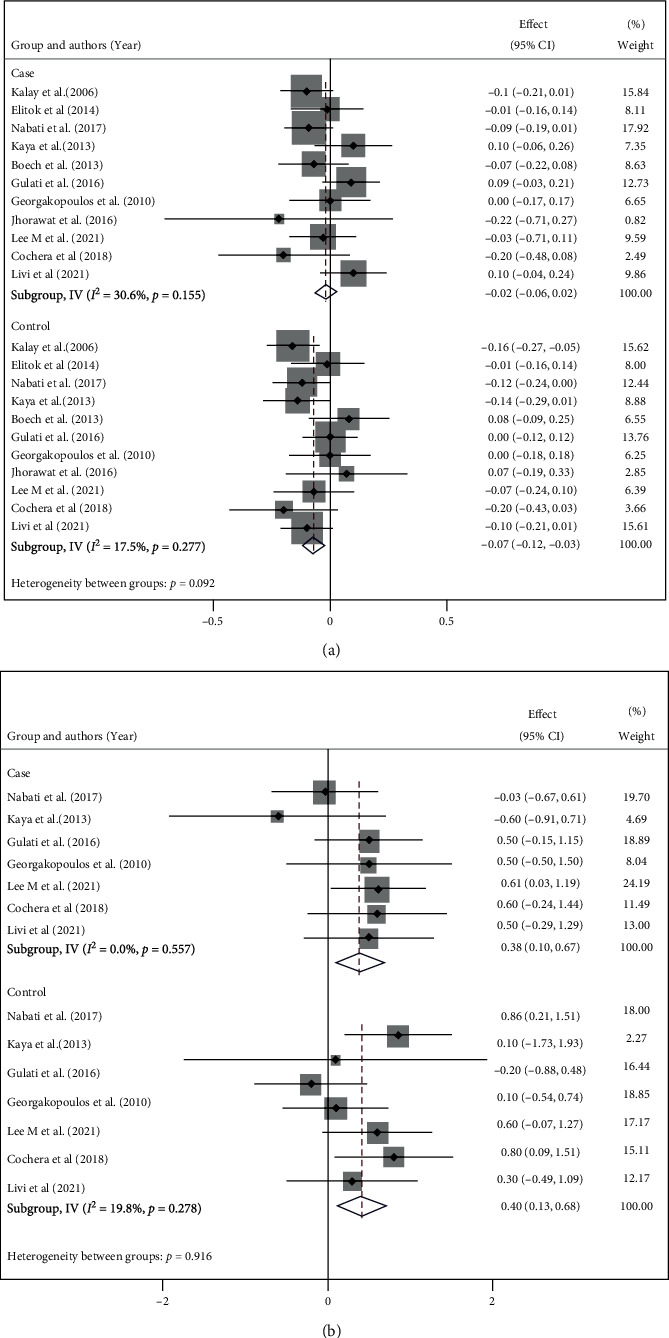
Forestplot of two group changes from the baseline of (a) E/A ratio and (b) E/e' ratio as an indices for diastolic function.

Several other echocardiographic parameters were described in the included studies. However, among these parameters, few addressed the interpretation of diastolic dysfunction. However, the E/A ratio was provided in eleven studies and was used to assess diastolic function. The overall MD of this ratio was calculated by fixed effect model, and the result in the intervention group was MD = −0.02 (95 CI%: -0.06, 0.02, *I*^2^ = 30.6%, *p* = 0.155); in the controls, it was MD = −0.07 (95% CI: -0.12, -0.03, *I*^2^ = 13.5%, *p* = 0.277; Figure 4(a)). E/e' ratio results are demonstrated in Figure 4(b). The other index representative of diastolic dysfunction is summarized in the supplementary Figure 6.

#### 3.4.6. Other Echocardiographic Measures

**Figure 5:**
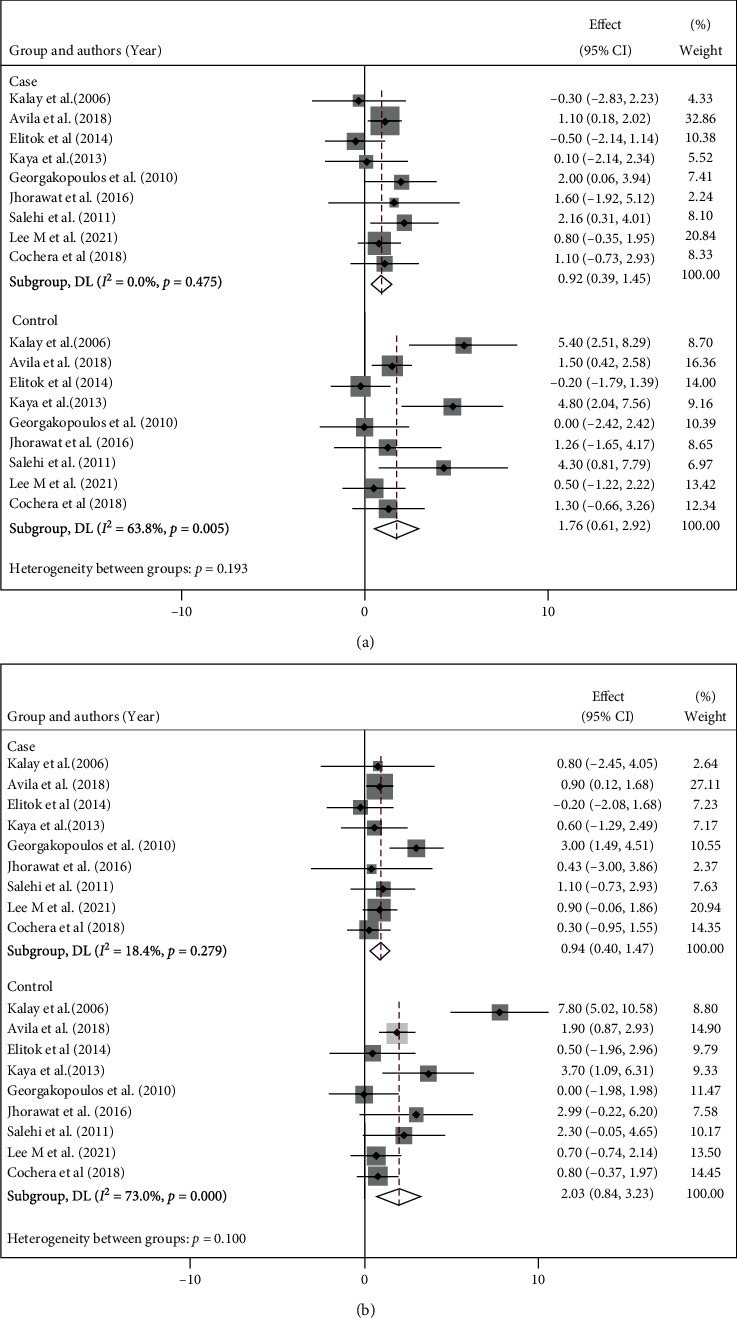
Forestplot of two group changes from the baseline in (a) Left ventricular end-diastolic diameter (LVEDD) and (b) Left ventricular end-systolic diameter (LVESD) LVEF.

Echocardiography was used not only for measuring LVEF but also for assessing the morphological and functional features of the heart. There are considerable discrepancies among the studies concerning different reported echocardiographic parameters. In this regard, eleven studies provided data on LVEDD and LVESD. Due to its difference in using combination therapy, one study was not included in the final analysis [16]. The LVEDD in the intervention and control groups were MD = 0.92 (95% CI: 0.39, 0.45, *I*^2^ = 0.0%, and *p* = 0.475) vs. MD = 0.1.76 (95% CI: 0.61, 2.92, *I*^2^ = 63.8%, and p = 0.005; Figure 5(a)), respectively. Likewise, LVESD was another parameter reported in the same studies as well. Pooling of data on this parameter showed a slight change from the baseline in the intervention group (MD = 0.94, 95% CI: 0.40, 1.47 *I*^2^ = 18.4%, and *p* = 0.279; Figure 5(b)); however, in the control group alternation of this parameter was more significant (MD = 2.03, 95% CI: 0.84, 0.3.23, I^2^ = 73.0% and p =0.001). The other reported echocardiographic finding is summarized in Supplementary Figures 6.

#### 3.4.7. Biomarkers

Three studies measured Troponin I as a representor of myocardial injury [6, 11, 15]. Since there was no study with zero troponin pathologic level value, this parameter's estimation was described by reporting RR. Therefore, based on the data provided by these three studies, the overall RR of this parameter was 0.68 (95% CI:0.49,0.95, *p* = 0.221, *I*^2^ = 33.7%) (Supplementary Figure 4).

B-type natriuretic peptide (BNP) was the other biomarker reported in three studies [6, 15, 23]. None of the biomarkers showed a significant difference between the intervention and control groups, and the results are presented in the supplementary file (Supplementary figure 5).

### 3.5. Risk of Bias Assessment

**Figure 6:**
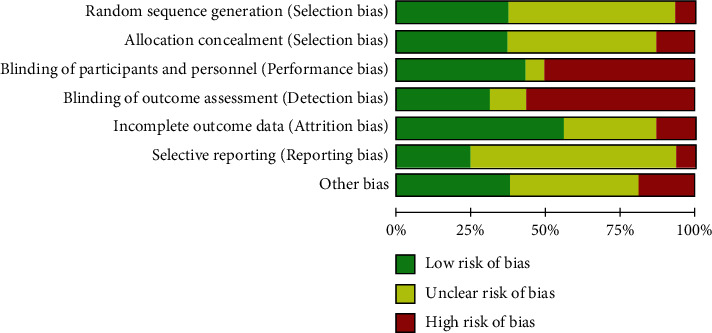
Risk of bias graph: review authors' judgments about each risk of bias item presented as percentages across all included studies.

Of all included studies, eight trials were open-labeled [9, 10, 12, 15–17, 20, 22], and of the remaining trials, three were placebo-controlled trials; therefore, they were considered single-blind [5, 11, 19] and there was another single-blind study which only the evaluator was blinded [14]. Moreover, five were double-blind due to mentioning the blinding of the outcome assessor and regarded method [6, 13, 18, 21]. Only four studies performed and described the method of Random sequence generation and allocation concealment simultaneously [6, 15, 18, 21], and four studies failed to describe the method of randomization and allocation [5, 16, 17, 19]. In Salehi et al.'s study [12], there were some substantial problems in reporting and describing the obtained results; therefore, we decided to consider other biases as high risk. Overall, we suppose three studies, as high quality [6, 18, 21] and the rest as unclear or low-quality trials. In one study, it was hard to judge accurately due to the nature of the publication type, a letter [17]. All included trials alleged proper baseline consistency and the criteria considered to exclude cases with conditions that possibly would influence the measurements or compliance (Figure 6).

## 4. Discussion

Here, we have shown that treatment with beta-blockers can prevent anthracycline-associated reduction in LVEF by 3.44%. However, this prevention does not translate into a significant reduction in the incidence of CTRCD and prevention of hospitalization for heart failure or cardiac death. To the best of our knowledge, by including 17 RCTs consisting of 1291 patients, this is the largest available study in the field.

Pooling the data of all 16 studies represents a reduction in LVEF in both intervention and control groups after chemotherapy (MD = −1.93% and MD = −4.78%, respectively). However, comparing the LVEF change between both groups showed that the LVEF was significantly less reduced among the beta-blocker receivers (MD = 3.44%, 95% CI: 1.41–5.46). Changes in other parameters of LV function, such as LVEDD and LVESD, have shown minimal change with beta-blocker therapy (0.92 mm and 1.76 mm for LVEDD, 0.94 mm vs 2.03 mm for LVESD, in intervention and control group, respectively). Our findings about changes in the LVEF are mostly dependent on the earlier components of the literature, with the recent PRADA and CECCY trials only exerting minimal influence on the pooled results. In the PRADA trial [18], metoprolol succinate was not effective in protecting against cardiotoxicity as a primary prevention measure. The study indicated a modest LVEF reduction in the no metoprolol (1.9%) and metoprolol (1.6%) groups [18]. In the CECCY trial, an even lower decrease was seen in the placebo (1.3%) and carvedilol (0.9%) groups [6]. Contrary to our results, several randomized trials have found carvedilol and nebivolol effective in preventing cardiotoxicity. This may be explained by limited sample sizes, higher doses of ANT, heterogeneity in the study populations (variations in risk factors, comorbidities, cancer status, and chemotherapy protocols), dissimilarities in study protocols and follow-up durations, and variations in techniques for determining the LVEF. The risk of cardiotoxicity is elevated among patients receiving a high cumulative ANT dose, as well as an increased number of risk factors [4]. Although variations were observed in the LVEF, all studies indicated the trend of a decrease in the LV end-diastolic diameter. By pooling the data, we found that LV chamber enlargement could be inhibited through beta-blockers, indicating the effect of carvedilol on LV remodeling in this context. In the PRADA trial [18], candesartan and metoprolol were assessed for their ability to avert the interstitial fibrosis associated with anthracycline therapy through T1 mapping and ECV, which correlated well with myocardial biopsy measurements [24].

In our study, diastolic function parameters were significantly more preserved with beta-blocker therapy. While the CECCY trial [6] revealed the beneficial effect of *β* blockers in averting diastolic dysfunction, inconsistent findings were obtained in two other trials [5, 18]. According to our data, no variations were seen across the two groups in parameters related to diastolic function other than the e' index. Accordingly, an association between beta-blockers and improvements in diastolic function cannot be ruled out. Indeed, based on the current guidelines, diastolic dysfunction is diagnosed according to the E/e', e', LA maximum volume index, and peak velocity of tricuspid regurgitation (TR) given a normal LVEF [25], and the LVEF of the participants of the included studies was >50%. Nonetheless, it should be mentioned that the mentioned studies did not measure the LA maximum volume and peak TR velocity indices. Notably, the result attained for the e' index was positive and that attained for the E/e' parameter was negative. Hence, specific conclusions can be made using these results. Therefore, we recommend that future studies should include all relevant parameters.

Our pooled analyses showed that 45 out of 370 participants in the intervention arm and 54 out of 341 participants in the control arm (from eleven studies) developed with CTRCD defined as a dramatic (more than 10%) reduction in LVEF (RR = 0.76; 95% CI: 0.53,1.09; *I*^2^ = 24.4%; *p* = 0.235). This finding does not accompany the beneficial finding of beta-blocker therapy on cardiac function indices such as LVEF. In fact, it needs to be clarified whether beta-blockers only impose beneficial hemodynamic effects or they may implement protective effects on the cardiac myocytes [4]. CTRCD has multifactorial pathophysiology. One of the key contributors is oxidative stress, caused by the generation of free-radical oxygen species due to interactions between doxorubicin and nicotinamide adenine dinucleotide dehydrogenase. As a result of oxidative stress, the integrity of the membranes of the cell and mitochondria is compromised, leading to myocardial cell injury and death. In an emerging theory, topoisomerase 2b inhibition has been implicated in the process of inducing the apoptosis of the cardiomyocytes [4]. Future experimental studies are needed to see if beta-blockers can affect any of these cascades, if true myocardial preservation happens or if just modification of the hemodynamic system is observed.

Our study had some limitations. Primarily, the review was limited to adult patients considering the variations between the adult and pediatric populations. The chief limitation, however, is the heterogeneous nature of the primary pooled data. This is a result of variations in methodology as well as differences in patient characteristics, including the breast cancer type/stage, level of immunocompetence, volume status, cardiovascular risk factors, underlying LV dysfunction, comorbidities, compliance, and disease predisposition. There is also unavoidable variability in the measurement techniques in the studies as the measured outcomes of echocardiograms are device dependent and subject to interobserver variability. Finally, we should also mention the fairly short (mean: 6 months) follow-up periods of the trials. Consequently, the rates of clinical events are very low, and the comparisons are weakened.

## 5. Conclusions

It can be concluded that treatment with beta-blockers has a statistically significant benefit in preventing a decline in cardiac systolic and diastolic function during anthracycline therapy which does not translate into a clinically significant reduction of the incidence of CTRCD, and in the prevention of hospitalization for heart failure or cardiac death. Therefore, routine administration of these medications for primary prevention of CTRCD cannot be recommended. Future investigations on selected high-risk populations, such as those with borderline primary LVEF or those receiving very high dosages of anthracyclines with a long duration of follow-up, are needed to see if these populations can gain a clinical benefit from such interventions or not.

## Figures and Tables

**Table 1:** Characteristics of the included studies.

Study	Number		Age(±SD) or (range)		Gender (female %)		Malignancy (%)	Beta-blocker regimen	ANT dosage(SD)		Radiation (%)		Primary end point	EF cut off
	IA	CA	IA	CA	IA	CA			IA	CA	IA	CA		
Kalay et al. [5]	25	25	46.8 ± 14	49.0 ± 9.8	22(88%)	21(84%)	Breast cancer 34 (68%) Lymphoma 9 (18%) Others 7 (14%)	Carvedilol	DOX 525.3 mg/m^2^ EPI 787.9 mg/m^2^	DOX 513.6 mg/m^2^ EPI 770.4 mg/m^2^	0	0	(1) Mortality (2) Echocardiographic parameters (3) Pulsed tissue Doppler imaging parameters (4) Troponin	LVEF <50%
Georgakopoulos et al. [17]	85	40	51.0 ± 18.0	49.1 ± 19.4	20 (48%)	19(47%)	Lymphoma 125 (100%)	Metoprolol	DOX 387.5 mg/m^2^	DOX 386.4 (5.7) mg/m^2^	8 (19)	9 (23)	(1) Echocardiographic parameters (2) Pulsed tissue Doppler imaging parameters	LVEF <50% LVEF drop >10%
Salehi et al. [12]	44	22	45.70 ± 14.16 52.52 ± 11.00	43.50 ± 15.27	32(72.2%)	14(63.6%)	Lymphoma 19 (28.8%) Breast 47 (71.2%)	Carvedilol	DOX 525.32 mg/m^2^ EPI 767.57 mg/m^2^	DOX 540.3 mg/m^2^ EPI 768.44 mg/m^2^	0	0	(1) Echocardiographic parameters (2) Pulsed tissue Doppler imaging parameters	NA
Liu et al. [16]	20	20	53 (39–68)	53 (37–65)	NA	NA	Breast cancer 40 (100%)	Carvedilol	NA	NA	NA	NA	(1) Electrocardiogram (2)Echocardiographic parameters (3)Troponin I	LVEF<45%
Kaya et al. [19]	27	18	51.4 ± 9.4	50.5 ± 11.1	100%	100%	Breast cancer 45(100%)	Nebivolol	DOX 257 (29) mg/m^2^ EPI 361(88) mg/m^2^	DOX 235(48) mg/m^2^ EPI 348(84) mg/m^2^	7 (26%)	5 (28%)	(1) Mortality (2) Echocardiographic parameters (3) Pulsed tissue Doppler imaging parameters (4) Troponin I (5) Plasma BNP	LVEF <45% LVEF drop >10%
Bosch et al. [15]	45	45	49.7 ± 13.9	50.9 ± 13.2	18 (40%)	21 (47%)	Acute leukemia 36 (40) Hodgkin lymphoma 9 (10) Non-Hodgkin lymphoma 23 (26) Multiple myeloma 22 (24)	Carvedilol	290^∗^(189) mg/m^2^	241^∗^(162) mg/m^2^	12 (27)	4 (9)	(1) Mortality (2) Echocardiographic parameters (3) CMRI (4) Pulsed tissue Doppler imaging parameters (5) Troponin I (6) Plasma BNP	LVEF <45% LVEF drop >10%
Elitok et al. [9]	40	40	54.3 ± 9.3	52.9 ± 11.2	40(100%)	40(100%)	Breast cancer 80 (100%)	Carvedilol	DOX 535.6 mg/m^2^	DOX 523.3 mg/m^2^	0	0	(1) Mortality (2) Echocardiographic parameters (3) Pulsed tissue Doppler imaging parameters	(1) LVEF drop >10% (2) LVEF drop>5% accompanying signs or symptoms of HF
Heck et al. [18]	62	58	50.5 ± 9.1	50.8 ± 9.2	62(100%)	58(100%)	Breast cancer 120(100%)	Metoprolol	NA	NA	45(72.5%)	40(66.7%)	(1) Mortality (2) Echocardiographic parameters (3) Pulsed tissue Doppler imaging parameters	(1) LVEF drop >10% (2) LVEF drop>5% accompanying signs or symptoms of HF (3) Death
Jhorawat et al. [10]	27	27	43.9 + 15.7	38.7 + _18.4	4(14.8%)	9(33.3%)	Lymphoma 53 (98) Acute leukemia 1 (2)	Carvedilol	DOX 267.36(76.126) mg/m^2^	DOX 252.65(77.82) mg/m^2^	0	0	(1) Mortality (2) Echocardiographic parameters (3) Pulsed tissue Doppler imaging parameters (4) Troponin I (5) Plasma BNP	LVEF <50% Drop >10%-30%
Tashakori et al. [13]	30	40	42.0(29 − 54)	39.9(29 − 54)	30(100%)	40(100%)	Breast cancer70(100%)	Carvedilol	DOX 240 mg/m^2^	DOX 240 mg/m^2^	0	0	(1) Echocardiographic parameters (2) Pulsed tissue Doppler imaging parameters	LVEF <50% Drop >10%-30%
Nabati et al. [11]	46	45	47.6 ± 8.7	47.1 ± 12.2	100%	100%	Breast cancer 91 (100)	Carvedilol	DOX 348.56 (40.34) mg/m^2^	DOX 359.91 (27.13) mg/m^2^	0	0	(1) Mortality (2) Echocardiographic parameters (3) Pulsed tissue Doppler imaging parameters (4) Troponin I	LVEF drop of >5% to <55% with HF LVEF drop of >10% to <55% without HF
Avila et al. [6]	96	96	50.8 ± 10.1	52.9 ± 9.05	96(100%)	96(100%)	Breast cancer 192 (100)	Carvedilol	DOX 240 mg/m^2^	DOX 240 mg/m^2^	0	0	(1)Mortality (2) Echocardiographic parameters (3) Pulsed tissue Doppler imaging parameters (4)Troponin I (5) Plasma BNP	LVEF <50% LVEF drop >10%
Lee et al. [14]	70	43	46.6 ± 7.6	45.8 ± 10.4	70(100%)	43(100%)	Breast cancer 113 (100)	Carvedilol	DOX 240 mg/m^2^	DOX 240 mg/m^2^	50(71.4%)	35(81.4%)	(1) Presence of DISC (2) Echocardiographic parameters	NA
Wihandono et al. [22]	26	25	44.5 ± 7.7	50.8 ± 7.39	26(100%)	25(100%)	Breast cancer 51 (100)	Lisinopril and Bisoprolol	DOX 579.48(65.10) mg	DOX 579.48 (65.10)mg	NA	NA	(1) Echocardiography paramenters	NA
Cochera et al. [20]	30	30	53 ± 13	52 ± 11	30(100%)	30(100%)	Breast cancer 60 (100)	Nebivolol	DOX 521(6) mg/m^2^	DOX 519(9) mg/m^2^	0	0	(1) Echocardiographic parameters	LVEF <50% LVEF drop >10%
Livi et al. [21]	45	42	48.8 ± 9.9	48.6 ± 7.9	42(100%)	45(100%)	Breast cancer 174(100%)	Bisoprolol	NA	NA	26(62%)	27(60%)	(1) Echocardiographic parameters	1.LVEF drop >10%

IA: interverion arm; CA: control arm; ANT: anthracyclines; DOX: doxorubicin; EPI: epirubicin: LVEF: left ventricular ejection fraction; HF: heart failure; NA: not available, DISC: doxorubicin-induced subclinical cardiotoxicity; BNP: brain natriuretic peptide. ^∗^The cumulative dose belongs to different anthracyclines.

## Data Availability

Data will be available based on request from the corresponding author.

## Supplementary Materials

Supplementary 1 PRISMA 2020 Checklist.

Supplementary 2 Supplementary Figure 1: forest plot of LVEF difference between both group at the end of the studies. Supplementary Figure 2: results of analysis of each study effect on pooled LVEF in beta-blocker receivers. Supplementary Figure 3: forest plots of mortality, development of cardiomyopathy, hospitalization, and development of heart failure. Supplementary Figure 4: forest plot of risk of developing pathologic troponin level. Supplementary Figure 5: forest plot mean difference (MD) and 95% confidence interval (CI) of BNP in intervention and placebo arms at the end of the study. Supplementary Figure 6: meta-analysis of echocardiographic parameters.
