# Supplementary material for: Beta-Blockers for Primary Prevention of Anthracycline-Induced Cardiac Toxicity: An Updated Meta-Analysis of Randomized Clinical Trials
Source: Cardiovasc Ther. 2022 Dec 29;2022:8367444. doi: 10.1155/2022/8367444 (PMC9818032; doi:10.1155/2022/8367444)
Supplement: Supplementary 2 — Supplementary Figure 1: forest plot of LVEF difference between both group at the end of the studies. Supplementary Figure 2: results of analysis of each study effect on pooled LVEF in beta-blocker receivers. Supplementary Figure 3: forest plots of mortality, development of cardiomyopathy, hospitalization, and development of heart failure. Supplementary Figure 4: forest plot of risk of developing pathologic troponin level. Supplementary Figure 5: forest plot mean difference (MD) and 95% confidence interval (CI) of BNP in intervention and placebo arms at the end of the study. Supplementary Figure 6: meta-analysis of echocardiographic parameters. [file 8367444.f2.docx]

**Supplementary materials:**


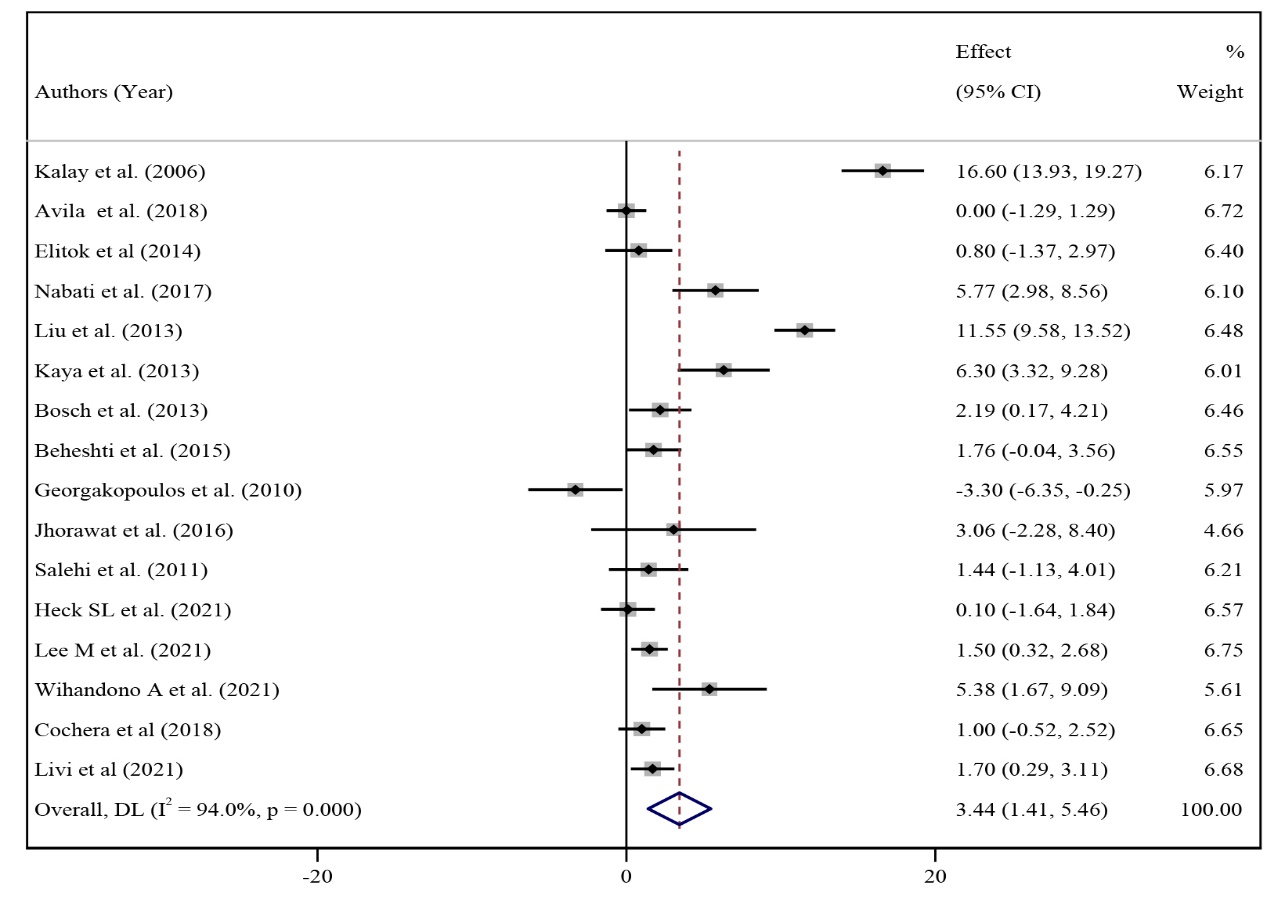


Supplementary Figure 1. Forest plot of LVEF difference between both group at the end of the studies.

Supplementary Figure 2. Results of analysis of each study effect on pooled LVEF in beta-blocker receivers


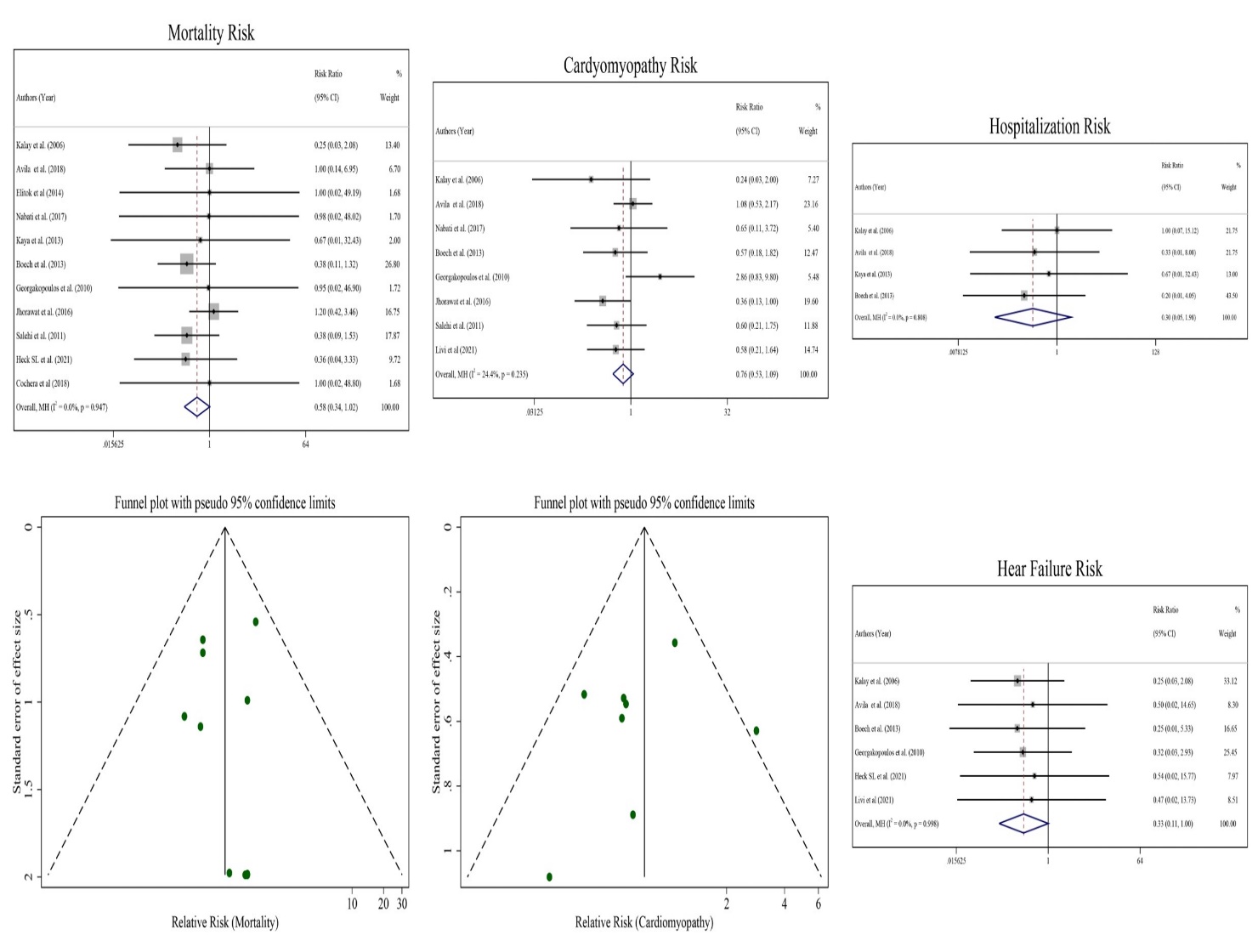


Supplementary Figure 3**.** Forest plots of mortality, development of cardiomyopathy, hospitalization and development of heart failure

**Results of biomarker analysis:**

Of all, four studies measured Troponin I as representor of myocardial injury. Bosch et al. reported that the overall degree of Troponin I rise in that study was mild [15]. Also, they could not find any relationship between the applied preventive regimen (carvedilol and enalapril) with LVEF and troponin changes (p=0.59) [15]. Likewise, according to Avila et al., the overall troponin raise was mild and hardly went above the upper limit which can imply the harmful, but undramatic, effect of ANT on the myocardium at the doses implemented in that study [6]. In a factorial trial using metoprolol and candesartan, it was reported that the level of troponin showed no difference between the metoprolol receivers and others [18]. Nabati et al. also reported the TnI level using intention to treat analysis. Unlike previous studies, they reported that the carvedilol could reduce the risk of ANT-induce cardiomyopathy [11]. The important thing about this study other than ITT analysis is that they used 6.25 mg dose of carvedilol twice a day which is a low dose of carvedilol compared to another study reporting the same marker and using the same preventive regimen [11]. Supplementary figure 4 showed the risk of developing pathologic troponin level.


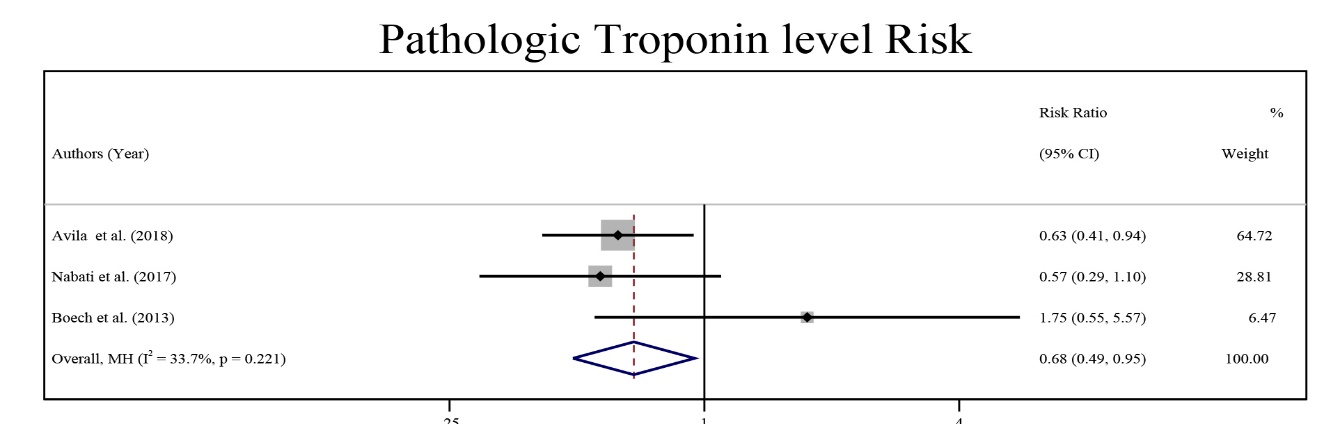


**Supplementary Figure 4.** Forest plot of risk of developing pathologic troponin level.

B-type natriuretic peptide (BNP) was the other biomarker reported in three studies [6, 15, 23]. Of these three studies, the data of two can be pooled for meta-analysis [6, 23]. The mean difference of BNP level from the baseline in the intervention group was 2.49 (95%CI= -8.86,13.84, I2=90.8% and p =0.001), and in the control arm it was -1.21 (95%CI= -4.15, 1,71, I2=0.0% and p= 0.350). (supplementary Figure 5).


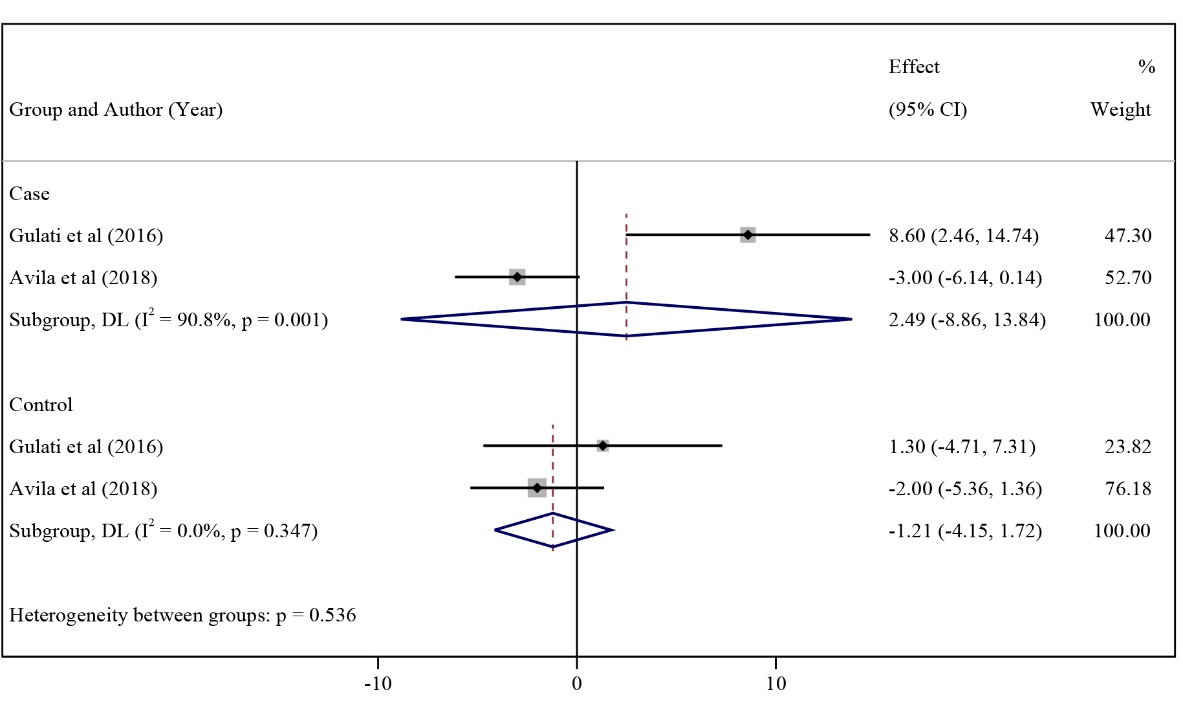


**Supplementary Figure 5.** Forest plot mean difference (MD) and 95% confidence interval (CI) of BNP in intervention and placebo arms at the end of the study.


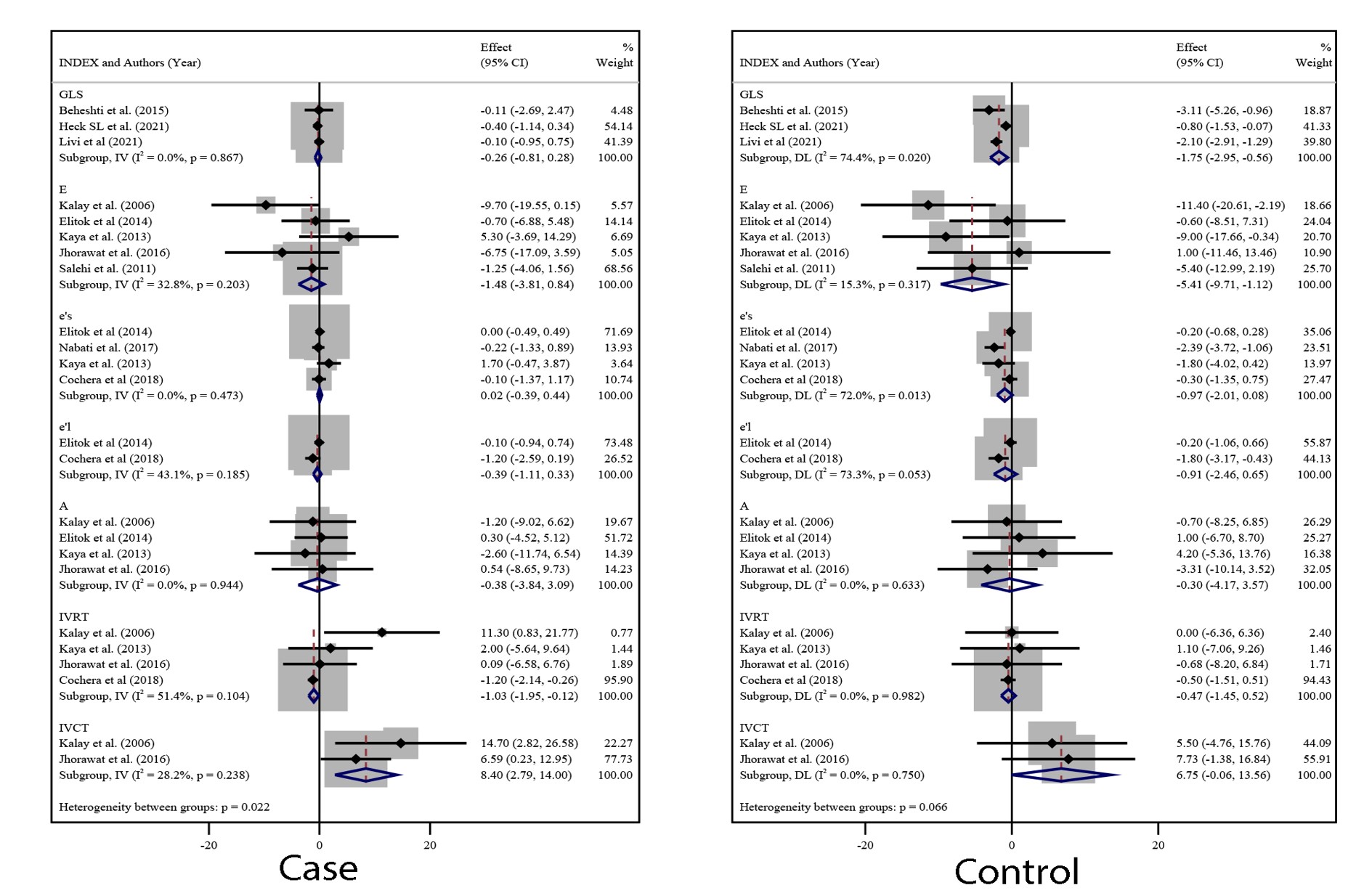


**Supplementary Figure 6.** Meta-analysis of echocardiographic parameters
